# Electronic Nose Based on an Optimized Competition Neural Network

**DOI:** 10.3390/s110505005

**Published:** 2011-05-04

**Authors:** Hong Men, Haiyan Liu, Yunpeng Pan, Lei Wang, Haiping Zhang

**Affiliations:** School of Automation Engineering, Northeast Dianli University, Jilin City 132012, China; E-Mails: lhyck01@126.com (H.L.); 187157331@qq.com (Y.P.); wanglei0510410@126.com (L.W.); zhplxzlq@tom.com (H.Z.)

**Keywords:** electronic nose, competitive neural networks, optimize

## Abstract

In view of the fact that there are disadvantages in that the class number must be determined in advance, the value of learning rates are hard to fix, *etc*., when using traditional competitive neural networks (CNNs) in electronic noses (E-noses), an optimized CNN method was presented. The optimized CNN was established on the basis of the optimum class number of samples according to the changes of the Davies and Bouldin (DB) value and it could increase, divide, or delete neurons in order to adjust the number of neurons automatically. Moreover, the learning rate changes according to the variety of training times of each sample. The traditional CNN and the optimized CNN were applied to five kinds of sorted vinegars with an E-nose. The results showed that optimized network structures could adjust the number of clusters dynamically and resulted in good classifications.

## 1. Introduction

The volatile odor of substances such as alcohol, tobacco, tea, food, *etc*. is closely linked to their quality. The electronic nose (EN) imitates an animal’s olfactory mechanism, which tests the volatile smell of food to detect the quality of certain foods. After their development over decades, ENs have become an objective and reliable tool for food quality testing applied to alcohol [1], fruits and vegetables [2], tea [3], meat [4] and other food industry products.

An EN is composed of a group of sensor arrays and some form of pattern recognition algorithm. The single sensor is unable to recognize certain complex odors. In order to increase the measuring accuracy of the sensors, researchers use gas sensors with partial selectivity to constitute an array and adopt an appropriate algorithm. Therefore, pattern recognition plays an important role in EN technology [5].

Presently, the pattern recognition algorithms which are applied to EN can be divided into two types—linear algorithms and nonlinear algorithms—according to the relationship between input variables and output variables. Examples of the former are k-nearest neighbor (k-NN) [6], linear discriminate analysis (LDA) [7–10], cluster analysis (CA) [11], principal component analysis (PCA) [12–17], Least Square Regression (LSR) [18–20] and of the latter, back propagation artificial neural network (BP-ANN) [21–23], probabilistic neural network (PNN) [24,25], Support Vector Machine [26], Radial Basis Function(RBF) [27], and self-organizing map (SOM) [28]. Among these algorithms, the neural network algorithm which is based on a biological neural network composition principle, with its self-organization, self-learning and parallel processing has been used widely in EN applications.

A competitive neural network (CNN) is a neural network clustering method. It has many merits like other neural network algorithms. Moreover, it has the merit that its learning algorithm is simple and fast. Consequently it is used widely in ENs. However, it also has many disadvantages as do most neural network algorithms:

1. It must determine the number of clusters first, namely fix the number of output neurons.
2. Once the network is successfully trained, the network will bear in mind the typical pattern. In the future, we can only use the network to identify these same types of samples. If a new sample is encountered, the sample can be attributed to its closest typical class. When the user cannot determine the number of samples in advance, the accuracy of competitive network identification results will be greatly reduced.
3. It selects initial weights randomly, and sometimes improper selection can lead to a slow convergence and incorrect sorting results.
4. The selection rule of the learning rate has a conflict between the convergence speed and the stability of the system.

These shortcomings restrict the application of the algorithm in electronic noses. For example, when evaluating the grade of tobacco, spices, and food freshness with electronic noses, the classification number of samples is not predictable and sometimes a new sample is not the same as the original samples stored in the network. However, it is also classified as one of them. Initial weights and learning rates that are selected randomly will make the electronic nose classification generate an undesirable result. In conclusion, there is a perceived need to improve the current competitive neural network algorithms in order to obtain more intelligent, and more practical ones.

This paper presents an open CNN structure which in terms of the DB value [29] determines the number of output neurons, specifically, the best number of clusters. The learning rate adjustment method and the selection of initial weights are also discussed. Finally, the optimized algorithm was applied to the classification of five kinds of vinegar with an EN, and the results showed that the network had a good dynamic classification; the network structure was stable, and quickly converged.

## 2. Experimental

### 2.1. Materials and Equipment

The experiment used five different kinds of vinegar samples: Zilin mature vinegar (ZiLin Food Co., Ltd.), Jiangcheng white vinegar (Jilin Brewing Industry Group Co., Ltd.), Lao Caichen aromatic vinegar (Lao Cai Chen Food Co., Ltd.), Liu Biju rice vinegar (Liu Biju Food Co., Ltd.), and Haitian fruit vinegar (Haitian Flavoring Food Co., Ltd.).

**Figure 1.**
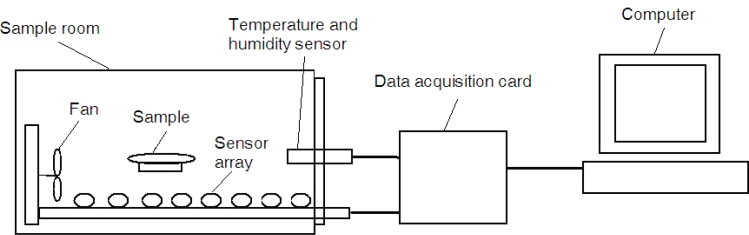
The electronic nose system.

In this study, a self-made EN system was used to test these vinegar samples. The core part was the gas sensor array, the specific sensor models included were: TGS 822, TGS 813, TGS 821, TGS 830, TGS 831, TGS 832, TGS 825, TGS 826 (produced by Tian Jin Figaro Electronic Co., China). The response signals of these sensors were in the 0∼5 V range, so the system did not need to amplify the signal. The sensor array was placed in the sample room. The sample room was a transparent 4,000 mL glass bottle, equipped with temperature-humidity sensor and gas mixing device. The system used an integrated HMT323 temperature and humidity sensor, which was produced by Vaisala Co. Its probe is small and flexible and thus easy to install. The measurement ranges of the sensor are: −40 °C to 80 °C, 0–100% RH. The gas mixing device used a small 1W fan to mix gas inside the room. The room was contained good air tightness so that various gas environments could be simulated. A typical data acquisition (DAQ) card iUSBU12086 (produced by HYIEK Automation Inc., Waterloo, Ontario, Canada) was employed as the A/D converter in the system. It can implement 8 Single-Ended, 12-Bit Analog Input Conversion, with a 32 k samples/s rate and a ≤0.1% conversion error. A schematic of the electronic nose is shown in Figure 1.

### 2.2. Experimental Methods

Before starting the equipment, the system needed to be preheated. When the response signals of these sensors were stable, we took a 10 mL sample of vinegar of each brand and put it into the evaporating dishes, in succession. Next we turned on the built-in fan to speed up the evaporation rate of the gas in order to make the gas concentrations in the sample room more uniform. In 40 seconds, the sensor signal has an obvious ascendant tendency; the data was collected and transferred to the computer through the data acquisition card. This data collection was maintained for 2 minutes, meanwhile, the switch of the fan was also controlled according to the data from the integrated temperature and humidity sensor lest any great change in temperature and humidity affect the results of the experiments. In the end, the data during the stable response was selected as the characteristic value. The characteristic value was first normalized, and then the data was put into the pattern recognition algorithm for classification. After each test, the system kept a fan on for a while, to reduce the adsorption of the previous sample on the sensor array and in order to prepare for the next test.

## 3. Competitive Neural Network

**Figure 2.**
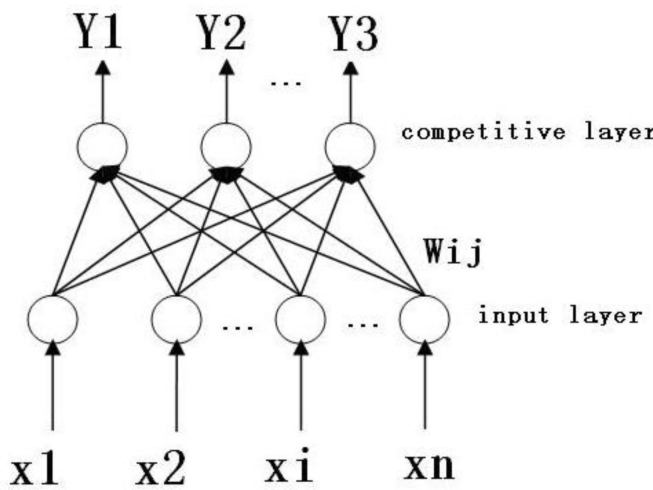
Structure of the competitive neural network.

Competitive neural networks imitate excitement, competition, inhibition and other mechanisms in biological neural networks to establish the network. The mode involves unsupervised network training, with parallel processing, simple learning algorithms, self-organization, and self-adaptive capacity, *etc*. The specific structure is shown in Figure 2.

The competitive neural network is composed of two layers. The first layer is the input layer; the number of neurons is the same as the dimension of input samples. The second layer is the output layer, also known as the competitive layer. The neurons in this layer are the same number as the kinds of the samples. The network structure has a two-way connection. The connective weights can be represented as W = (w_ij_, i = 1, 2, ... m; j = 1, 2, ..., n), where w_ij_ represents the competitive weight of the input neuron i and the competitive neuron j. The specific learning methods are:

1. Confirm the specific network structure: fix the number of the neurons in the input layer and the competitive layer, and then, the weights and the learning rate are assigned the random numbers in [0, 1] as the initial value.
2. Supposing that the data of the input sample is vector: X = [×1, ×2... ×n]^T^, we can calculate the Euclidean distance for all neurons in two layers:
  (1) $$d(W_{i},\;X)={\left[{\left(W_{i}-X\right)}^{T}\;(W_{i}-X)\right]}^{2}$$
3. The output neuron which has the minimum value is the winner. Then the weights which are connected to it are adjusted to a favorable direction for its future success. This is seen in Formula (2):
  (2) $$w_{ij}^{\prime }=w_{ij}+\eta (x_{j}-w_{ij})$$
4. Calculate the value of the error function *Et : Et* = Σ[*w*(*t*) –*w*(*t +* 1)]^2^, if the value is less than the given threshold, the training is stopped, otherwise return to Step (2), until it meets the minimum error value.

**Figure 3.**
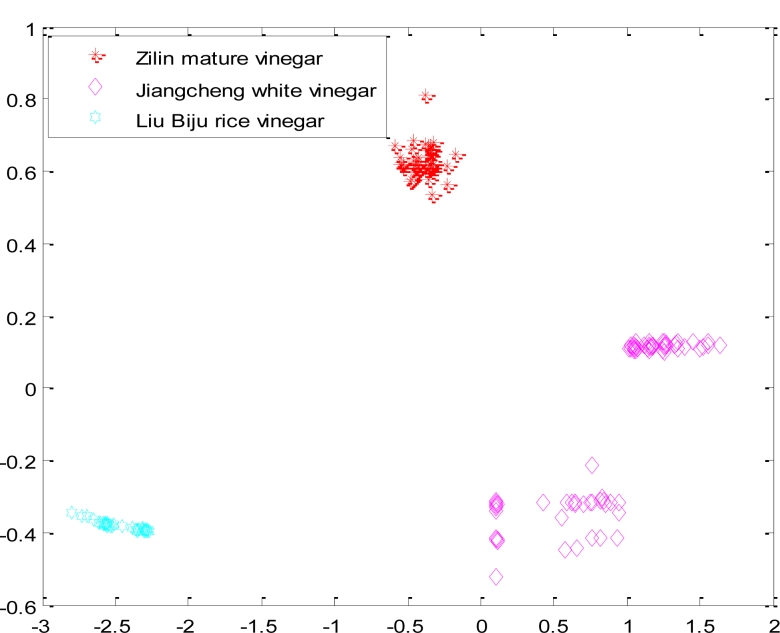
The result of classifying four kind samples with a traditional CNN.

Ultimately, each network layer weight vector of neurons is adjusted to the nearest value of a certain type of input vectors. When the test sample is put in, the network will attribute it to the closest of its kind. According to the experimental data, we selected the feature data of four kinds of samples (Zilin mature vinegar, Jiangcheng white vinegar, Lao Caichen aromatic vinegar, Liu Biju rice vinegar.), then put the data into the traditional competition in order to be classified. Figure 3 is the result when the network used random initial weight values, took a fixed learning rate *η* =  0.4, and the given number of output neurons was four. Figure 3 shows how the test samples of Zilin mature vinegar, Jiangcheng white vinegar, Lao Caichen aromatic vinegar and Liu Biju rice vinegar were put into the network. The samples of Lao Caichen aromatic vinegar and Jiangcheng white vinegar were judged as the same sample.

**Figure 4.**
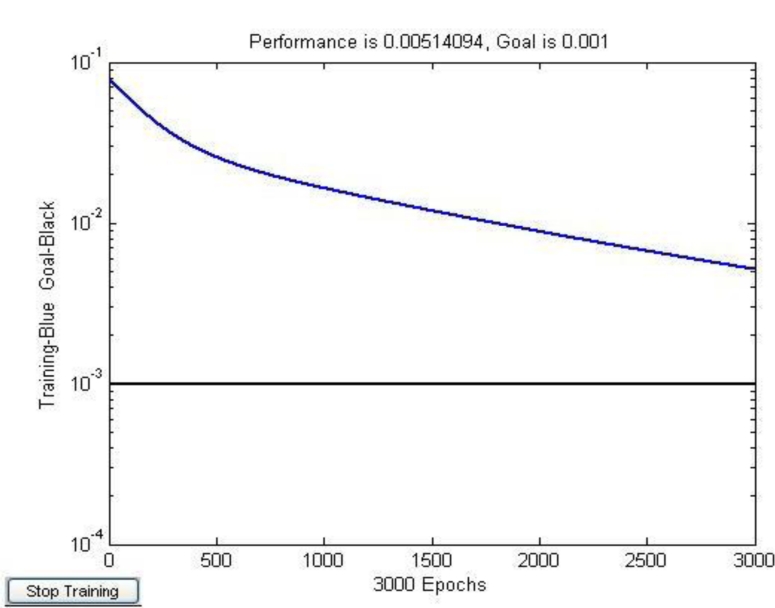
Error convergence of the traditional CNN.

Figure 4 shows the error convergence of the traditional CNN, because the initial weight and the learning rate were selected randomly, the value of error function dropped extremely slowly, even if the training times reached the maximum (3,000), it still could not achieve the objective error *ɛ* =  0.001 .

**Figure 5.**
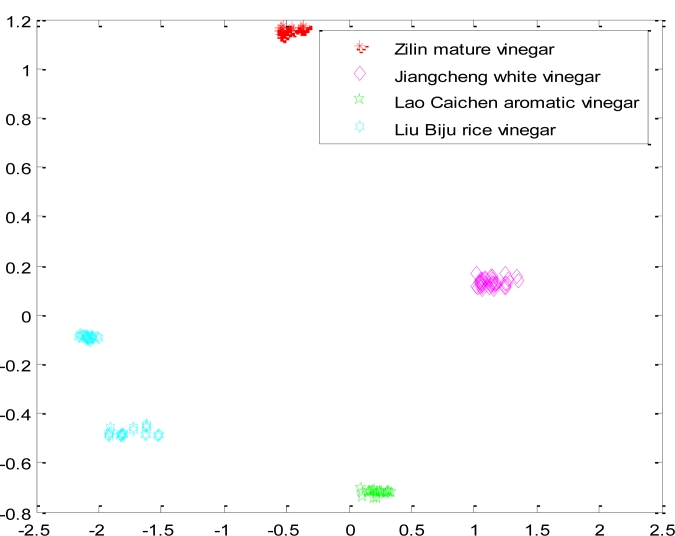
The result of classifying the fifth sample with the traditional CNN.

The initial weight and the learning rate were adjusted until the network could classify the samples correctly. After saving the adjusted network, the fifth sample (Haitian fruit vinegar) was identified. The sample was not recognized as a new class, but rather was classified into the Liu Biju rice vinegar group as shown in Figure 5.

## 4. Method of Optimizing the Competitive Neural Network

To optimize it this paper presents an open competitive neural network architecture style. The specific learning methods are as follows.

### 4.1. Confirm Initial Connection Weights

Firstly, the initial connection weights have a great influence on the convergence and learning rates. If the learning vector is a finite part of the whole pattern space, while the connection weights are distributed randomly in all directions, there will be many differences between the input and the weight vectors which will result in the convergence rate slowing or not converging. Therefore, this design gave all w_ij_ (i = 1, 2, ... m; j = 1, 2, ... n) the same initial value. Because of this, the initial values are close to the normalized characteristic values of each sample, thus reducing the time of the input vector selecting the weight vector in the initial stage to enhance the rate of adjustment of the weight vector.

### 4.2. Adjustment of Learning Rate

Learning rate *η* refers to the rate of change of connecting weight vectors to the input sample. Learning rate affects the training results of the network greatly. According to the results of a large number of experiments, it is known that if *η* is too small, it will result in the convergence rate slowing, however, if it is too big, it will cause the structure of the network to become unsteady, so we made *η*a function, shown in Equation 3, as follows: it can make *η* small in the beginning stage, with the training times increasing *η* augmented slowly step by step, in the end, the value of *η* is decreased gradually.

(3) $$\eta =\text{sin}[2\pi \times (\frac{t-fix(\frac{t}{c})\times c}{\frac{T}{N}})]$$

where *t* is for the current training times, *c* is the number of training required for each sample, *T* is the total times for the training, *N* is the number of categories for the current sample. The result of the experiment shows that the adjustment method of the learning rate not only could stabilize the structure of the network but also could ensure fast convergence.

### 4.3. Adjust the Number of Neurons

Here we introduced the DB value which was proposed by Davies and Bouldin and used to determine the optimal clustering of a number. The specific definition of DB is:

(4) $$DB=\frac{1}{n}\sum_{i=1,i\neq j}^{n}\text{max}(\frac{d_{i}+d_{j}}{d(c_{i},\;c_{j})})$$

where n is the number of clusters, d_i (j)_ is the average distance between class i (j) samples and their cluster centers c_i (j)_, d (c_i_, c_j_) is the distance between the cluster center c_i_ and c_j_. The cluster center of each class is the farther, and the most effective is the better. When the DB value reaches the minimum, the classification effect is the best.

The most appropriate number of output neurons is determined according to the DB values, then merging, splitting or deleting the output neurons can occur. The concrete method is executed as follows:

1. The method of merging neurons is that the comparability of the weight vectors is computed first. If the value of the comparability exceeds a certain threshold, we merge the two output neurons. The comparability is calculated as follows:
  (5) $$L_{i}(w_{i},\;w_{j})=\frac{w_{i}\times w_{j}}{∥w_{i}∥\times ∥w_{j}∥}$$
  (6) $$∥w_{i}∥=\sqrt{w_{i1}^{2}+w_{i2}^{2}+\ldots +w_{in}^{2}}$$
  (7) $$∥w_{j}∥=\sqrt{w_{j1}^{2}+w_{j2}^{2}+\ldots +w_{jn}^{2}}$$
  The weights of merged neuron are showed as follows:
  (8) $$w_{\text{new}}=\frac{1}{n_{i}+n_{j}}(n_{i}w_{i}+n_{j}w_{j})$$
  where n_i_ and n_j_ represent the number of i samples and j samples, respectively.
2. The method of splitting neurons is that the split neuron which has the largest volume of super ball [30] into two neurons; the ball’s volume is computed by Formula (9):
  (9) $$V_{k}=\sqrt{\frac{1}{n_{k}}\sum_{i}{\left(x_{i}-w_{k}\right)}^{T}\;(x_{i}-w_{k})}$$
  where k is the category mark of x_i_. On the assumption that w_m_ is selected to be split, the new divided neuron weight vector w_m1_ and w_m2_ are shown as:
  (10) $$w_{m1}=w_{m}+\theta \sigma_{k}$$
  (11) $$w_{m2}=w_{m}-\theta \sigma_{k}$$
  where *θ* is an empirical constant in (0,1), *σ_k_* is the variance vector of splitting neurons.
3. The method of deleting the neuron is to remove the one which doesn’t have any samples, then moving the other neurons to new locations, and the number of clusters decreases.
4. When the network structure is stored, and a new sample is added, firstly, we compute the comparability between the new sample and former sample as seen in Formula 5, if the value of the comparability exceeds a certain threshold, determine it to belong to this kind, otherwise, add a new neuron:
  (12) $$w_{\text{new}}=[0,0,\ldots 0]+x_{\text{new}}$$

### 4.4. Main Steps of Optimized Competitive Neural Network

The main steps of the optimized structure of neural network are as follows:

1. Give the number of output neurons the initial value N.
2. Set values of initial weight *w_ij_*.
3. The samples are classified by the traditional competitive neural network. Firstly, compute the comparability between the sample and any weights, if the values are all very small, increase a new neuron as Formula (12), and then go to Step (3), otherwise, go to Step (4).
4. If the output neurons do not have a corresponding sample, delete the node and reduce the number of output neurons, then repeat Steps (3), otherwise go to Step (5)
5. Calculate the value of the current DB (k). If *DB*(*k*) –*DB*(*k –*1) > *α* (*α* is empirical value), then go to Step (6). Otherwise calculate the error function of each weight, if it reaches the threshold value, the algorithm will stop, otherwise, go to Step (3).
6. Calculate the comparability among the weights of all neurons, if the comparability is greater than the threshold, combine neurons as seen in Formula (8), and the number of category is reduced 1, after that go to Step (3), otherwise, go to Step (7).
7. Calculate the volume of super ball, and choose the largest one to split the node according to Formula (10), (11), and the number of categories is reduced 1, and then go to step (3).

## 5. Application of the Optimized Competitive Neural Network

**Figure 6.**
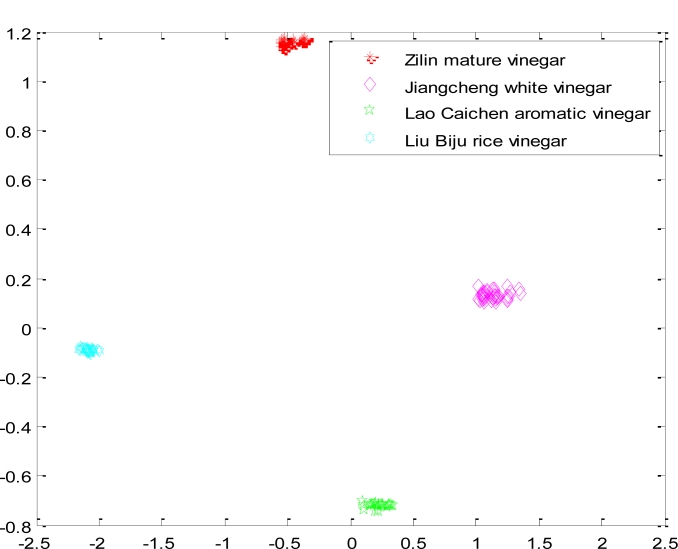
The result of classifying four kinds of samples with the optimized CNN.

Set the parameters *θ* = 0.1, *ɛ* = 0.001, comparability *λ* =  0.65, threshold of DB *α* =  0.028, initial number of clusters N = 2, the total training time *T* = 3000. Because the parameters have been changed, the network structure is entirely different from the previous traditional CNN, that this, it is a new network. In succession, we confirmed the validity of the optimized CNN as follows:

First, we selected four kind samples (Zilin mature vinegar, Jiangcheng white vinegar, Lao Caichen aromatic vinegar, Liu Biju rice vinegar), the same as the traditional CNN, and put their feature data into the optimized network. The samples were separated completely. The results are shown in Figure 6.

**Figure 7.**
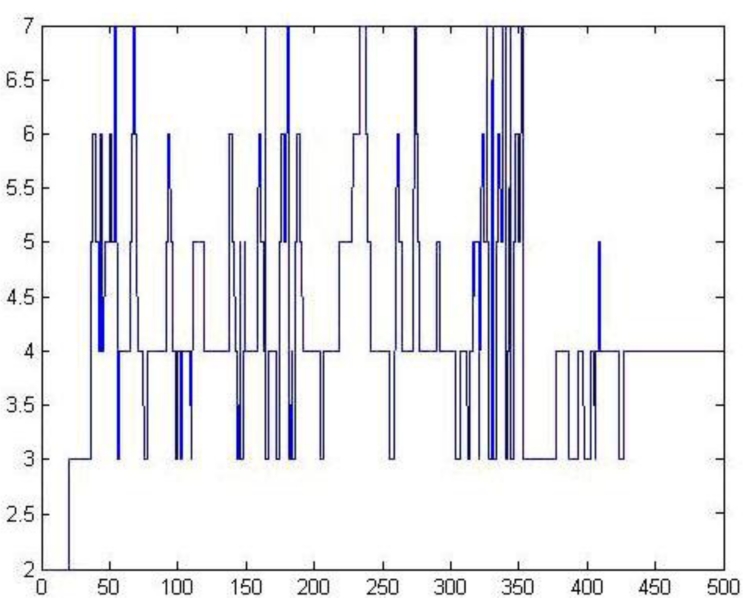
The variation of output nodes.

The number variation of the output nodes is shown in Figure 7. It shows that during the number of output nodes, four was the maximum density, finally, the number of output nodes is stably four, indicating that the optimized network can adjust well to the number of categories.

**Figure 8.**
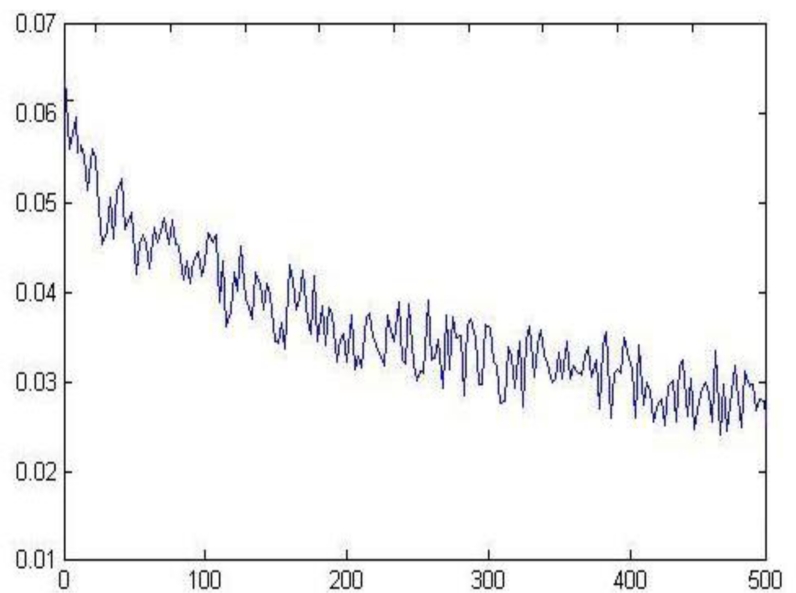
The adjustment of DB value.

Figure 8 shows that the DB value achieved a stable minimum through continuous adjustment, namely, the number of categories was the most reasonable at this time.

**Figure 9.**
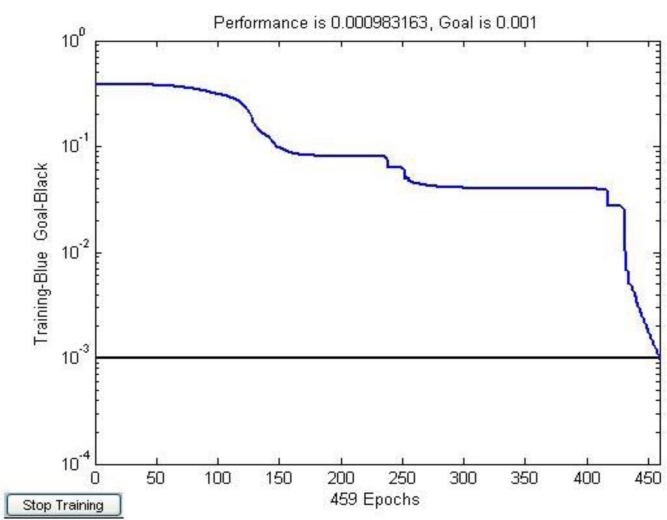
The error convergence of the optimized CNN.

Figure 9 shows that the optimized learning rate was changed along with the training times. The revised direction was corrected in the training process, which made the value of error function decrease rapidly, and the training objective was reached in step 459. The training speed of the optimized network was much faster than the traditional CNN (Figure 4).

**Figure 10.**
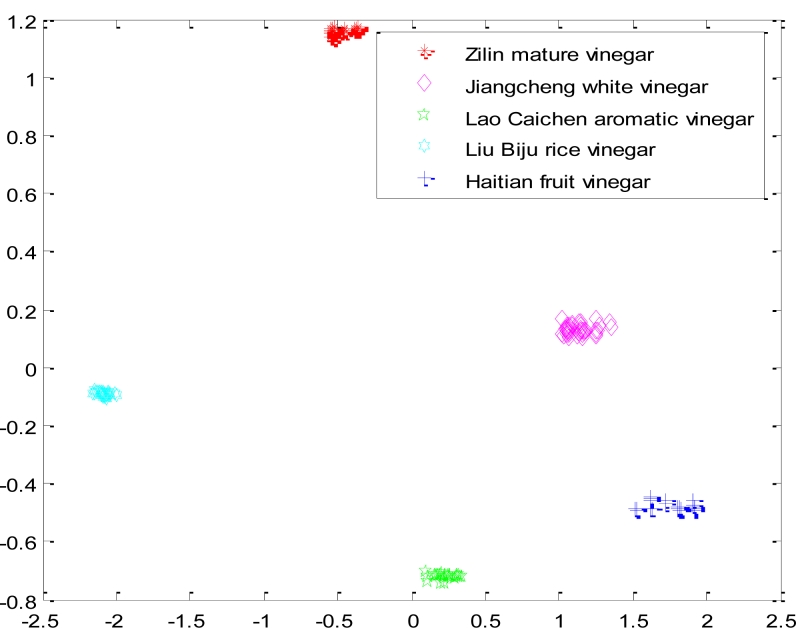
The result of classifying the fifth sample with the optimized CNN.

After saving the network, the fifth sample (Haitian fruit vinegar) was identified. The results are shown in Figure 10. The new sample was correctly determined as a new category with the optimized network by judging the comparability, so the optimization purpose was reached.

**Figure 11.**
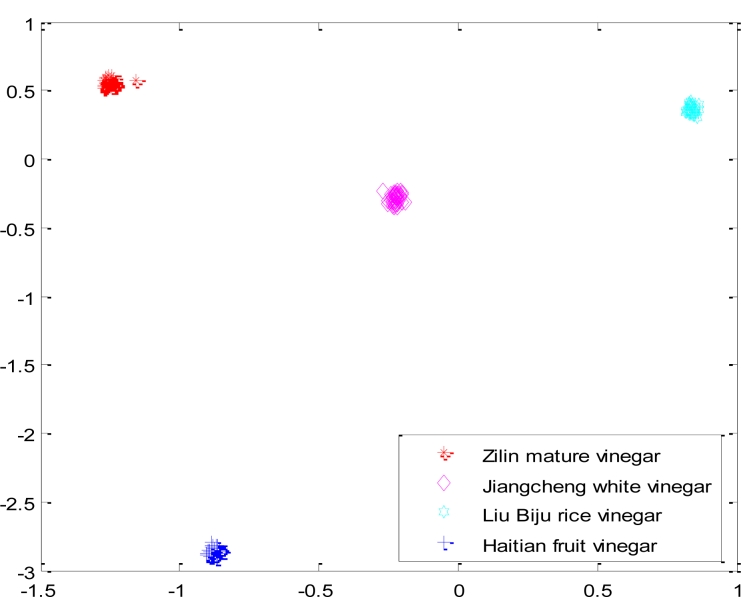
The result of classifying a new set of four samples with the optimized CNN.

In order to verify the stability and the reliability of the network, we made a repeated independent measurement trial with a new set of samples (Zilin mature vinegar, Jiangcheng white vinegar, Liu Biju rice vinegar Haitian fruit vinegar), and the classifying result is shown as Figure 11.

**Figure 12.**
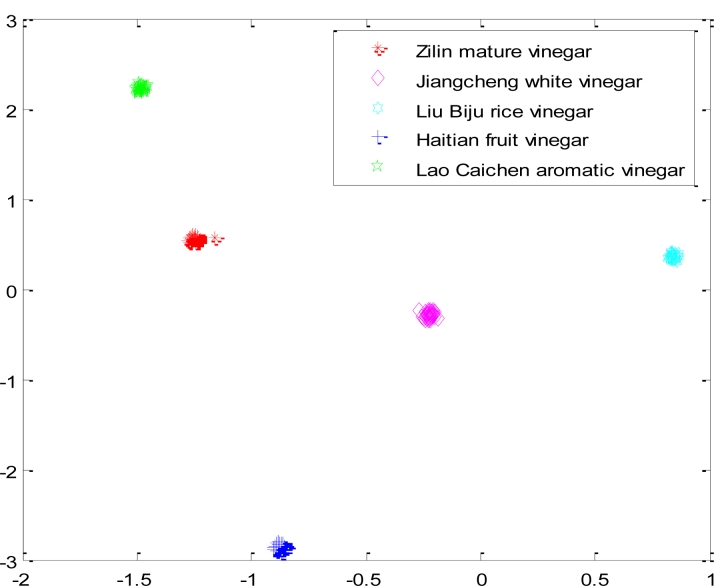
The result of classifying a new set of four samples with the optimized CNN.

After the network was saved, we put the fifth sample (Lao Caichen aromatic vinegar) into the network as seen above, and the classifying result is shown in Figure 12. We can see the performance of the e-nose coupled to the Optimized Competitive Neural Network method is good.

## 6. Conclusions

The paper introduces an optimized competition neural network implemented by setting the initial weights, adjusting the learning rates, and adjusting the number of neurons according to the DB value. The optimized CNN was used to recognize the vinegar samples with EN and received a good classification effect. Therefore, the optimized algorithm can be applied in EN and make EN more intelligent.
